# Micropapillary: A component more likely to harbour heterogeneous *EGFR* mutations in lung adenocarcinomas

**DOI:** 10.1038/srep23755

**Published:** 2016-04-05

**Authors:** Yi-Ran Cai, Yu-Jie Dong, Hong-Bo Wu, Zi-Chen Liu, Li-Juan Zhou, Dan Su, Xue-Jing Chen, Li Zhang, Ying-Li Zhao

**Affiliations:** 1 Department of Pathology, Beijing Chest Hospital, Capital Medical University, Beijing Tuberculosis and Thoracic Tumour Research Institute, 97 Beiguan Machang Rd, Tongzhou District, Beijing, 101149, P.R. China; 2 Department of Medical Oncology, 1st Division, Beijing Chest Hospital, Capital Medical University, Beijing Tuberculosis and Thoracic Tumour Research Institute, 97 Beiguan Machang Rd, Tongzhou District, Beijing, 101149, P.R. China

## Abstract

The micropapillary (MP) subtype has recently been established to be a distinct marker of poor prognosis in lung adenocarcinomas (LACs). According to the 2015 WHO classification system, LAC constituents are required to be precisely reported. T790M mutation and an insertion in exon 20 (E20ins) are associated with *EGFR*-TKI resistance. A total of 211 LAC patients were involved in this study, and *EGFR* mutations were determined using an amplification refractory mutation system (ARMS). Sex, smoking history, lymph node status, and clinical stage differed significantly between the *EGFR* wild type and mutant groups (p < 0.05). The *EGFR* mutation occurred more frequently in female, non-smokers, ACs with papillary (85.7%) or MP components (91.4%) (p < 0.001). Twenty ACs with naïve T790M or E20ins were microdissected. The AC constituents metastasizing to lymph nodes exhibited a phenotype and EGFR status that was consistent with the primary loci constituents. Glomerulus-like solid components exhibited the same *EGFR* status as the surrounding T790M-mutated MP components. The MP and glomerulus-like portions in AC tumours exhibited a congenial *EGFR* status, but the acinar cells with papillary cells were heterogeneous. The naïve T790M mutants, although minor in the MP component, dramatically increased after *EGFR*-TKI therapy and indicate that the MP components feature intrinsic heterogeneity.

Adenocarcinoma (AC) is one of the most common histological types of non-small cell lung cancer (NSCLC)^1^. More than 80% of lung ACs are diagnosed based on tumour constituent mixtures in accordance with the 2004 WHO classification system. New classification systems that better considered the proportions of AC components were then established by the International Association for the Study of Lung Cancer (IASL), the American Thoracic Society (ATS) and the European Respiratory Society (ERS) in 2011^2^, as well as the WHO classification system in 2015^3^. These new classification systems aim to classify invasive adenocarcinoma histological subtypes according to their precise components, such as lepidic (formerly the most mixed tumour subtype with nonmucinous BAC), mucinous (formerly mucinous BAC), acinar, papillary, solid patterns and micropapillary (MP) ACs. These systems have demonstrated an improved ability to address complex histological phenotypes and improve molecular and prognostic correlations^4^. Recent studies of stage IB lung ACs report that micropapillary ACs are predominant and are the most common AC subtype with an *EGFR* mutation, whereas predominantly solid ACs present with a lower frequency^5^. Specifically, the MP subtype is associated with worse outcomes^6^,^7^. Notably, several recent studies have noted that the MP pattern may have a clinical impact on patient survival rates^8^,^9^. According to the new WHO classification system, all components in the bulk of a tumour are required to be listed in precise proportions. In another study, 20 out of 21 (95.2%) MP-predominant lung adenocarcinomas harboured *EGFR* (85.7%) driver mutations. MP-predominant ACs are more likely to recur than ACs harbouring MP components (>5%) in stage I lung AC^10^.

*EGFR* mutations occurring between exon 18 and exon 21 are associated with a response to tyrosine kinase inhibitors (TKIs), and *EGFR* mutations are mainly categorized as activating (frame deletion in exon 19 and L858R) or TKI-resistant (T790M and insertions in exon 20) mutations^11^,^12^,^13^. The T790M mutation was initially considered to be a secondary mutation following progression after *EGFR*-TKI therapy, but several recent reports have suggested that T790M exists at a low frequency before *EGFR*-TKI therapy^14^,^15^. Moreover, using highly sensitive methods, studies have shown that T790M frequencies range from 40% to 79%, even in *EGFR*-TKI-naïve NSCLC patients harbouring *EGFR*-sensitive mutations^16^,^17^,^18^. These results suggest that *EGFR*-mutated tumours may intrinsically include a minor population of T790M-positive cancer cells. At an advanced stage, lung cancers are inoperable, and targeted therapy is critical for such patients. Therefore, the main issue for modern biomedical research is understanding cellular and molecular mechanisms. Because the association between morphological diversity and biological heterogeneity in ‘mixed’ ACs remains unclear, understanding the relationship between histological and biological attributes in AC components is critical. In our previous study, we used a high-resolution melt (HRM) curve to screen *EGFR* statuses in AC primary and metastasized tumours and found different *EGFR* statuses among these tumours (Fig. 1). Because laser capture microdissection (LCM) enables researchers to combine structural identification with molecular investigation, these methods aid in investigating pathological changes on a molecular, cellular, or tissue level^19^. The purpose of the current study is to analyse the correlation between de novo *EGFR*-TKI-resistant mutations and histological diversity in AC subtypes.

## Results

### Patient Characteristics and Histopathological Features

**Figure 1:**
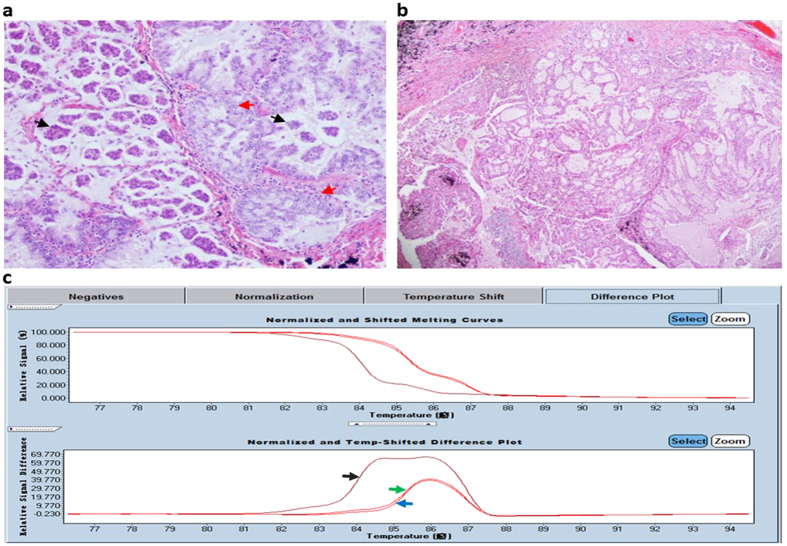
Exon 19 status of the *EGFR* gene was screened using high-resolution melting curves in the primary and metastasized tumours of a ‘mixed’ AC. The ‘mixed’ AC was composed of micropapillary (black arrow) and acinar (red arrow) patterns in the primary locus (**a**, ×200), whereas only acinar cells occupied the metastasized lymph node and destroyed its normal structure (**b**, ×40). An HRM analysis of the *EGFR* status shows that the primary lesion curve (black arrow) exhibited a different character compared with a metastasized tumour (green arrow) and peripheral normal tissue (blue arrow)(**c**).

Clinicopathological features are summarized in Table 1. A total of 211 patients with invasive AC were observed; invasive AC was slightly more common in females (51.7%, n = 109) than in males. The median age was 61 years for the females (range, 34–87 years) and 60 years for the males (range, 33–82 years). Of the 211 patients, 142 (67.3%) had never smoke. Eighty-eight cases (41.7%) were stage I or II; 84 (39.8%) were stage IIIA; and 39 (18.5%) were stage IIIB or IV. Fifty-eight (27.5%) and 98 (46.4%) ACs featured MP or papillary patterns, respectively. Furthermore, 29.8% of the cases were diagnosed of as pure papillary AC, and 11.8% cases were diagnosed as pure MP AC. Of the 135 ACs with local lymph node invasion, 74.1% (43/58) of tumours harbouring an MP pattern exhibited more aggressive growth (*p* = 0.04, one sided comparison). The other ACs contained solid, acinar, lepidic or mucinous components. We analysed the primary ACs and metastasized AC phenotypes (Fig. 1), and the metastasized neoplastic cells featured one or more of the patterns in the primary loci.

### Histological Subtypes and *EGFR* Mutation Analysis

Of the 211 AC tumours, 117 (55.5%) featured an *EGFR*-sensitive mutation (deletion in the exon 19 frame, or L858R), and 67 were *EGFR* wild typed. The T790M and insertion mutations in exon 20 (E20ins) were the *EGFR*-TKI-resistant mutations identified in our study. Twenty ACs (9.5%) featured a de novo *EGFR*-TKI-resistant mutation, either with a sensitive mutation (7.1%) or independently (2.3%). Six ACs (2.8%) featured less frequent mutations, such as G719X, L861Q and S768I. Only one AC included both a frame deletion in exon 19 (E19del) and L858R. The presence of *EGFR* mutations significantly correlated with gender and smoking status, especially in females (78.9%, *p* < 0.001), non-smokers (76.8%, *p* < 0.001) and histological subtypes with MP (91.4%, *p* < 0.001) and papillary components (83.7%, *p* < 0.001). Ninety percent of the 30 ACs with both papillary and MP components as the predominant components included an *EGFR* mutation. Similarly, ACs with a predominant pattern with either papillary or MP components also exhibited a higher *EGFR* mutation incidence (83.3% and 92.3%, respectively) than ACs with acinar (60.6%) and lepidic (20%) components. Local lymph nodes were invaded in 135 (64%) ACs. Ninety-seven (68.3%) ACs with an *EGFR* mutation and 43 (74.1%) ACs with a MP pattern were susceptible to lymph node invasion (*p* = 0.04, *p* = 0.04, respectively).

### Naïve *EGFR*-resistant mutations in AC components in both primary and metastasized tumours determined using a HRM curve and ARMS

**Figure 2:**
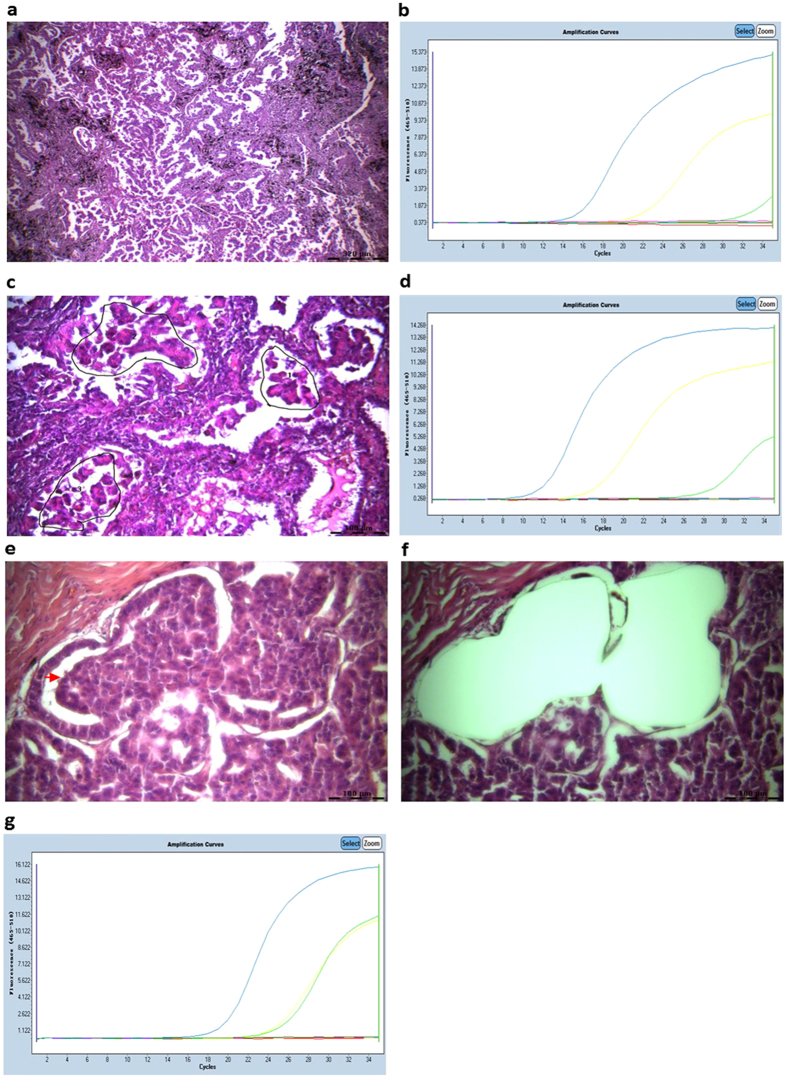
Different histological portions of an adenocarcinoma consisting of micropapillary and solid components (patient No 154) were laser microdissected, and *EGFR* mutants were determined using ARMS. In the micropapillary portion, small clusters of glandular cells lacking fibrovascular cores grow within an airspace covered by cancer cells (**a**) and the *EGFR* status of the tumour mass was demonstrated using L858R (yellow curve) and T790M (green curve) mutations (**b**). Micropapillary cells were separated by laser microdissection (**c**), and L858R and T790M were found together (**d**). In the solid area, malignant cells are woven in tufts as a glomerulus-like pattern (red arrow), and the tumour cells are crowded in a solid or plate structure (**e**) and were captured using LMD (**f**). The *EGFR* mutation status was the same as that of the micropapillary cells (**g**).

Twenty ACs with naïve *EGFR*-TKI resistance were investigated to analyse molecular heterogeneity and phenotype. The specific histological components from the primary and metastasized foci are listed in Table 2. In this study, the sensitive mutations were more likely to accompany the T790M mutation. Eighty-five percent (17/20) of the tumours exhibited papillary or MP architecture. Furthermore, ACs harbouring MP (30%) and papillary (76.9%; 10/13) components were observed with the T790M mutation. In addition, E20ins was detected in 5 ACs, 4 of which were mixed ACs and only one was a pure papillary AC. The acinar pattern was predisposed to E20ins (60%; 3/5). Four ACs were investigated with solid, acinar or lepidic structures and E20ins. Seventeen ACs (85%) were mixed with more than one pattern, and the sensitive and resistant mutation curve cycle threshold (Ct) values differed (Fig. 2). Therefore, we used LMD to discern different morphological tissues and to investigate the *EGFR* status of the heterogeneous components. The ARMS amplification curves show that the T790M mutation was prevalent in papillary (76.9%; 10/13) and MP (100%; 6/6) components with or without a sensitive mutation (Fig. 2a,b). The discrepant Ct values for each component show that the papillary or MP components exhibited intrinsic heterogeneity (Fig. 2c,d). Furthermore, 53.8% (7/13) of the metastasized tumours exhibited one or more phenotypes of the primary loci (Table 2). An ARMS amplification revealed that the *EGFR* status of the metastasized cells was consistent with the corresponding primary tumour components.

**Figure 3:**
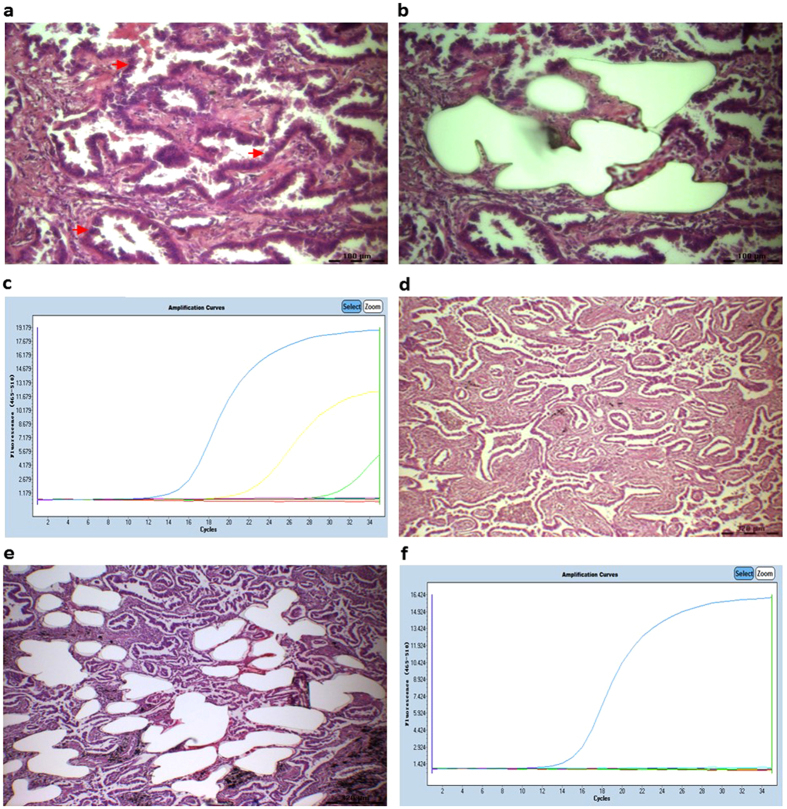
An adenocarcinoma composed of papillary and acinar components (patient No. 144) was laser microdissected, and the *EGFR* mutations were investigated in different histological components. Cuboidal to columnar cells grow in a layer along fibrovascular cores with papillary pattern. (**a**) Tumour cells were captured (**b**) and we determined their composite L858R (yellow curve) and T790M (green curve) mutations (**c**). Acinar cells did not exhibit the same shape as papillary cells but a glandular pattern (**d**). Laser-captured cells (**e**) also featured a wild-type *EGFR* status, which differed from the papillary cells (**f**).

Solid or plate patterns exhibited varied *EGFR* statuses in different tumour masses. The solid structure coexisted with MP, papillary or even acinar components. A solid structure with papillary or MP components was often accompanied by architectures such as ‘glomerulus’ spreading and transforming to adjacent MP or papillary patterns. The glomerulus-like cell tufts featured both the same cytological features and *EGFR* status, which indicates that these tufts are woven micropapillae or papillae with a fissure around a lumen (Fig. 2e–g). Furthermore, papillary cells accompanied by MP cells in the bulk of the tumour exhibited the same cytological characteristics. The histological arrangements showed cuboidal or columnar cells growing on a fibrovascular core with an apical cytoplasm protruding into the airspace, similar to a ‘hobnail’. This form was conspicuous and unusual with a conventional papillary pattern (Fig. 3a–c). Acinar cells with papillary cells did not exhibit the same shape as the papillary cells, and molecular heterogeneity was observed when these tumour cells were mixed in a tumour mass (Fig. 3d–f).

**Figure 4:**
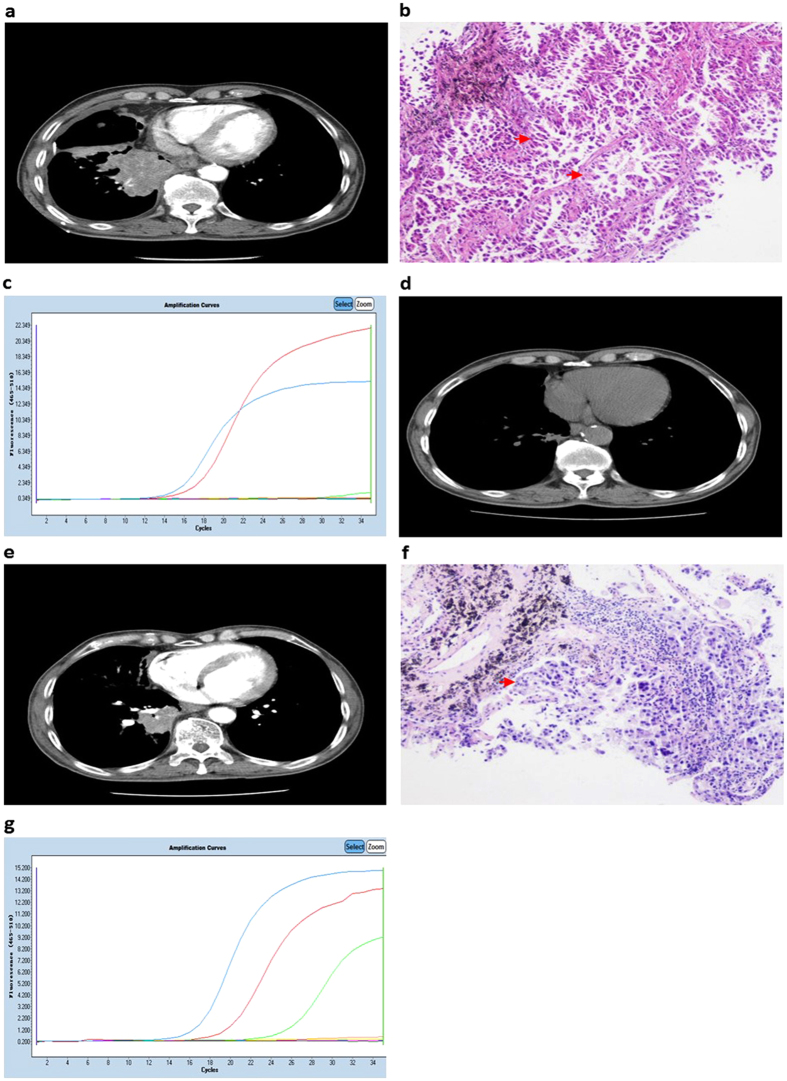
A patient was used as an example to clarify the heterogeneity of the micropapillary AC. A tumour mass was present in the inferior lobe of the right lung, which was determined using computed tomography (**a**) and the histological type was a pure micropapillary type (**b**). An ARMS analysis of the *EGFR* status showed a deletion in exon 19 (red curve) and a minor naïve T790M mutation (green curve) (**c**). Conspicuous inhibition was observed using computer tomography after 9 months of *EGFR* TKI therapy (**d**). However, progression of the tumour was observed after 11 months of therapy (**e**) one more biopsy was performed, and the histological type remained the micropapillary type but with poorer differentiation (**f**). The T790M mutant had clearly increased in the micropapillary tissue (green curve) (**g**).

In the current study, one MP AC patient progressed to an advanced stage with metastasis to bilateral lung, invasion to parietal layer of pleura and right supraclavicular lymph nodes (T4N3M1a) (Fig. 4a) and then received a needle aspiration on the primary locus. The histological type was MP (Fig. 4b), and the *EGFR* mutation test using ARMS showed an E19del mutation despite a minute quantity of T790M (for the manual criteria, a positive T790M requires that the Ct value is ≤28 cycles) (Fig. 4c). Tumour size obviously shrank after 9 months Iressa therapy (Fig. 4d). However, the tumour began to progress again (Fig. 4e), and one more biopsy was performed. The tumour histological pattern persisted on the MP components, the newly progressed tumour exhibited poor differentiation (Fig. 4f), and the T790M mutation clearly increased with the E19del (Fig. 4g). This case exhibits the innate heterogeneity of MP ACs.

## Discussion

Increasing evidence shows that histological subtype is a prognostic factor that is independent of *EGFR* mutations^20^. Articles on the topic of MP ACs have reported patients with a poor prognosis, even at an early stage^21^,^22^. A recent study demonstrated that the MP pattern ratio correlated with the TNM stage and lymph node metastasis. Moreover, MP predominant subtypes are more likely to occur with *EGFR*-mut tumours in Chinese lung AC patients^23^. Our results also show that *EGFR* mutation incidence is more frequent in ACs with MP (91.4%) and papillary cells (85.7%), and ACs with *EGFR* mutations prone to local lymph nodes were observed for the MP components. The MP and solid subtypes are common in tumours past stage I, sizes >2.5 cm and with a pure solid type, which are predictors of poor DFS. The MP subtype is a single prognostic factor for OS^9^. In this study, MP, papillary and a glomerulus-like solid structure coexisted, and neoplastic cells with a solid area grew in tufts without a fibrovascular core and were crowded in a lumen. In addition, the cells appear as MP cells. The same *EGFR* status in the MP and solid portions of a tumour mass suggested that they may have the same origin and response to *EGFR*-TKI. Molecular alterations of *EGFR*, *KRAS*, and *BRAF* genes are more frequent in MP adenocarcinoma than in other pulmonary adenocarcinoma subtypes^21^. These alterations should be studied in low papillary areas to discern their relationship with MP ACs. Typically, MP cells appear detached and/or connected to alveolar walls lacking a fibrovascular core. Nevertheless, the papillary cells in this study exhibited a clear cellular pattern with the cytoplasm protruding into a glandular cavity, which was the characteristic growth pattern for the MP cells. In light of the similarities between the low papillary and MP patterns in lung adenocarcinomas, can we hypothesize that low papillary areas are early precursors of MP carcinoma and a harbinger for later MP carcinoma development? We compared *EGFR* statuses and demonstrated that papillary cells with a TKI-resistant mutation feature the same status as adjacent MP cells and even metastasized loci.

Intratumoural *EGFR* homogeneity in lung cancers has long been assumed. Herein, a patient’s *EGFR* mutation status was interpreted using a qualitative method, such as sequencing. However, only a fraction of patients harbouring *EGFR* mutations respond to *EGFR*-TKIs; thus, additional factors beyond an *EGFR*-sensitive mutation contribute to a patient’s drug response. In addition to morphological diversity, the potential for remarkable tumour heterogeneity represents a major challenge to personalized medicine and biomarker development^24^. In the clinical scenario, it could explain the mixed responses to targeted therapies^25^. Several related biomarkers (e.g., T790M, K-ras, and C-met) have been suspected in *EGFR*-TKI resistance. Based on recent studies, intratumoural *EGFR* mutational heterogeneity is a candidate mediator for *EGFR*-TKI therapy resistance; however, the existence of such heterogeneity remains controversial^26^,^27^. The mutations T790M and E20ins are notable resistance mutations and associated with a secondary mutation after *EGFR*-TKI therapy^11^. In the current study, sixteen ‘mixed’ ACs harboured sensitive and/or TKI-resistant mutations in addition to 4 histologically pure ACs. Because the primary T790M mutation featured different Ct values than the coexistent sensitive mutation, the LMD and ARMS methods were introduced to analyse the molecular heterogeneity and morphological diversity. The outcomes show that T790M was prevalent in the papillary and MP components, whereas E20ins was more common in the acinar components. However, solid patterns were also observed with acinar patterns in the bulk of the tumour. Cells in the solid area featured an abundant cytoplasm similar to acinar components and harboured the same *EGFR* status as acinar cells. These outcomes suggest that solid components are paralogous constituents with poor differentiation. In the current study, the *EGFR* statuses were compared between primary and metastasized foci. Out of the 20 ACs with a TKI-resistant mutation, 13 were metastasized, with at least one component from the primary foci, and featured the same mutation type as the primary tumour. We suspected that these ACs may respond to *EGFR*-TKI with the same efficacy. However, acinar cells with papillary or MP components exhibited a different *EGFR* status and a different predominant form. Therefore, the acinar cells were considered a heterogeneous subclone in the tumour cell population. Considering these presentations, we noted that the MP and papillary cells were the most heterogeneous components in lung ACs. Fukutomi *et al.* reported that MP tumours and low papillary patterns should be differentiated based on morphology to avoid confusing terminology. In brief, a low papillary structure was observed in the lepidic components, whereas a MP pattern was observed in the nonlepidic components. We also observed an extensive lepidic growth pattern in the peripheral area of low papillary cells, especially adjacent to normal lung tissue. *EGFR* status revealed a congeneric relationship between low papillary and lepidic cells. A low papillary structure is a significant histological feature of the lepidic component and is associated with aggressive cancer behaviour in lung adenocarcinoma^28^.

Indeed, heterogeneity between primary and metastatic tumours for a limited number of markers (hormonal receptors and HER2 status in breast cancer) has been associated with markedly worse outcomes^29^,^30^. Whether these results reflect inappropriate use of targeted therapies or more aggressive tumours with increased genomic instability that produces multiple subclones has not been determined. In our study, a patient at an advanced stage featured a E19del mutation and a minute quantity of T790M without the positive criterion (Ct value ≤28) and received Iressa therapy for 10 months. New progression was observed using computed tomography. Another biopsy was performed, and the *EGFR* status was determined as an E19del mutation with increasing T790M mutations. The MP pattern persisted with poorer differentiation than before the TKI therapy. This example shows that MP components feature a histological pattern with innate heterogeneity.

Our study suggests that it is critical to determine the heterogeneity of AC components, such as solid, papillary and MP components, by their EGFR status because such components are common in histological subtypes with worse prognoses. Other constituents, such as acinar, may exhibit different molecular features than coexisting papillary or MP cells. Intrinsic heterogeneity is a common biological attribute in MP cells and is an EGFR-TKI resistance mechanism.

## Materials and Methods

Excised invasive lung adenocarcinomas were collected at Beijing Chest Hospital from 2013 to 2015. All sample sections were evaluated by two pathologists (YR. Cai and YJ. Dong) to confirm the diagnoses using the 2015 WHO classification criteria and a multi-headed microscope. A mean number of 4.5 slides (range, 1–11 slides) were reviewed, and representative tumour sections were simultaneously determined for further analyses. Histological patterns were calculated though the precise proportions of the components. The clinical stage was defined in accordance with the 7th Edition of the TNM Classification of the Union for International Cancer Control^2^,^31^.

### DNA extraction, polymerase chain reaction amplification and *EGFR* mutation assay

Fifty milligrams of tumour tissue from tumour masses was scraped off representative formalin-fixed and paraffin embedded (FFPE) blocks. The *EGFR* gene status was tested using an amplification refractory mutation system (ARMS) (AmoyDx Inc. Xiamen, China). The genomic DNA extraction and *EGFR* status assay were performed in accordance with the manufacturer’s protocols (AmoyDx Inc. Xiamen, China).

### Diverse phenotypic components of lung adenocarcinoma determined through laser microdissection and *EGFR* status determined through high-resolution melt curves and ARMS

Typical sections were selected from each specimen both in primary and metastasized loci and were stained with haematoxylin and eosin. Two 6 μm-thick sections were used for laser microdissection (LMD) using a Leica LMD7000 (Leica microsystems, Wetzlar, Germany). Notably, cancer cells with a histological pattern were as pure as possible. If a tumour tissue consisted of cancer cells with different *EGFR* mutation states, we could determine the relationship between the histological constituents and the genetic attributes. No less than 500 tumour cells from each distinctly histological component were collected, and the DNA was purified using a QIAamp Micro Kit (Cat. No. 56304, Germany) following the manufacturer’s protocol^32^. Because the genomic DNA quantity was extremely limited in our study, we used high-resolution melt curves (HRMs) to screen the *EGFR* status, and ARMS was used to determine the mutation type. We used an easy-to-use reaction mix for PCR and a HRM kit (Cat No. 04909631001, Roche, Germany) with a Cobas z 480 PCR amplifier following the manufacturer’s manual with previously described primers^33^. We compared the HRM curve shapes to screen *EGFR* status and evaluated the quantity though the cycle threshold value via ARMS.

Ethics approval was obtained from the ethics committee of Beijing Chest Hospital, Capital Medical University, Beijing Tuberculosis and Thoracic Tumour Research Institute and the informed consent was obtained from all subjects. The methods were performed in accordance with the approved procedures from the corresponding manufacturers.

### Statistical Analysis

Clinicopathological factors were compared using crosstab χ^2^ or Fisher’s exact test. We used one-sided and two-sided statistical tests, and the significance level was α = 0.05. All statistical analyses were performed using the JMP system for Windows (version 11, SAS Institute Inc, Cary, NC).

## Additional Information

**How to cite this article**: Cai, Y.-R. *et al.* Micropapillary: A component more likely to harbour heterogeneous *EGFR* mutations in lung adenocarcinomas. *Sci. Rep.* **6**, 23755; doi: 10.1038/srep23755 (2016).

## Figures and Tables

**Table 1:** Clinicopathological characteristics and *EGFR* status in 211 AC patients.

		*EGFR* status					
Clinical characteristics		Wild type (%)	Mutation (%)	*p*value	Sensitive mutation (deletion in exon 19 or L858R) (%)	Naïve TKI-resistant mutation (E20 ins & T790M) (%)	Other mutations (G719X, L861Q) (%)
Gender	Female	22 (20.2)	87 (79.8)	0.0002	71 (65.1)	14 (12.8)	2 (1.8)
	Male	45 (44.1)	57 (55.9)		47 (46.1)	6 (5.9)	4 (3.9)
Smoking history	Smoked	36 (52.2)	33 (47.8)	<0.001	28 (40.6)	4 (5.8)	1 (1.4)
	Never smoked	32 (22.5)	110 (77.5)		89 (63.4)	16 (11.3)	5 (3.5)
Clinical stage	I + II	38 (43.2)	50 (56.8)	0.001	37 (42.1)	12 (13.6)	1 (1.1)
	IIIa	15 (18.1)	68 (81.9)		59 (71.2)	5 (6)	4 (4.8)
	IIIb + IV	15 (37.5)	25 (62.5)		22 (55)	3 (7.5)	1 (2.5)
Histological components	with MP	5 (8.6)	53 (91.4)	<0.001	46 (79.4)	5 (8.6)	2 (3.4)
	without MP	62 (40.5)	91 (59.5)		72 (47.1)	15 (9.8)	4 (2.6)
	with papillary	14 (14.3)	84 (85.7)	<0.001	68 (69.4)	12 (12.2)	4 (4.1)
	without papillary	53 (46.9)	60 (53.1)		50 (44.3)	8 (7)	2 (1.8)
LN invasion	Yes	36 (26.7)	99 (73.3)	0.034	85 (62.9)	10 (7.4)	4 (3)
	No	31 (40.8)	45 (59.2)		33 (43.4)	10 (13.2)	2 (2.6)

MP, micropapillary. LN, lymph node. E20ins, insertions in exon 20 of the *EGFR* gene.

**Table 2:** Histological components and naïve *EGFR*-TKI resistant mutations in primary and metastasized tumours.

Patient No.	TNM/Clinical stage	Histological components and *EGFR* mutation					
		Histological components in primary loci	Histological components in LNs	*EGFR* mutation type	Histological patterns in primary loci (*EGFR* mutation type)	Histological patterns in metastasized locus (Mutation type)	
6	T1N2 M0/IIIA	80% solid, 20% acinar	Solid and acinar	E20 ins	solid (E20 ins), acinar (E20 ins)	solid (E20 ins), acinar (E20 ins)	
11	T1N1 M0/II	100% MP	MP	L858R & T790M	MP (L858R & T790M)	MP (L858R & T790M)	
50#	T2aN2 M0/IIIA	90% papillary, 10% lepidic	–	Del in E19 & T790M	Papillary and lepidic (Del in E19 & T790M)	–	
52*	T4N2 M0/IIIB	70% acinar, 30% papillary	papillary	Del in E19 & T790M	Papillary (Del in E19 & T790M); acinar (WT)	Papillary (Del in E19 & T790M)	
55*	T2aN2 M0/IIIA	60% papillary, 40% acinar	papillary	E20 ins	Papillary (WT); acinar (E20 ins)	Papillary (WT)	
71	T2bN0 M0/IIA	95% papillary, 5% MP	–	Del in E19 & T790M	Papillary (Del in E19 & T790M); MP (Del in E19 & T790M)	–	
72	T2N1 M1/IV	100% papillary	papillary	E20 ins	Papillary (E20 ins)	Papillary (E20 ins)	
93	T3N2 M0/IIIA	80% solid, 20% acinar	solid, acinar	E20 ins	Solid (E20 ins); acinar (E20 ins)	Solid (E20 ins); acinar (E20 ins)	
112	T1aN1M0/IIA	80% solid, 20% MP	solid	L858R & T790M	Solid (L858R & T790M); MP (L858R & T790M)	Solid (L858R & T790M)	
120	T1aN0 M0/IA	70% papillary, 30% MP	–	L858R & T790M	Papillary (L858R & T790M); MP (L858R & T790M)	–	
144*	T1bN1M0/IIA	40% acinar, 60% papillary	papillary	L858R & T790M	Acinar (WT); papillary (L858R & T790M)	Papillary (L858R & T790M)	
150	T2aN2 M0/IIIA	60% solid, 40% acinar	Solid, acinar	Del in E19 & T790M	Solid and acinar (Del in E19&T790M)	Solid and acinar (Del in E19 & T790M)	
154	T1bN1 M0/IIA	60% solid, 40% MP	MP	L858R & T790M	Solid (L858R & T790M); MP (L858R & T790M)	MP (L858R & T790M)	
160*	T2N2 M1/IV	70% papillary, 30% acinar	papillary	Del in E19 & T790M	Papillary (Del in E19& T790M); acinar (WT)	Papillary (Del in E19 & T790M)	
166*	T1bN0 M0/IA	70% solid, 20% acinar, 10% papillary	–	L858R & T790M	Solid and papillary (L858R & T790M); acinar (WT)	–	
171	T1aN0 M0/IA	lepidic	–	E20 ins	Lepidic (E20 ins)	–	
174	T2aN0 M0/IB	60% acinar, 30% papillary, 10% solid	–	Del in E19&E20 ins	Acinar (E20 ins); papillary & solid (Del in E19)	–	
194	T1bN0 M0/IA	40% papillary, 30% acinar, 30% lepidic	–	L858R & T790M	Papillary & lepidic (L858R & T790M); acinar (WT)	–	
197	T1bN0 M0/IA	60% papillary, 40% lepidic	–	Del in E19 & T790M	Papillary & lepidic (Del in E19 & T790M)	–	
199	T1bN1 M0/IIA	100% MP	MP	Del in exon19 & T790M	MP (Del in E19 & T790M)	MP (Dele in E19 & T790M)	
202	T2bN2 M0/IIIA	95% acinar, 5% papillary	acinar	L858R & T790M	Acinar (L858R & T790M); papillary (L858R & T790M)	Acinar (L858R & T790M)	

^*^patients with a discrepant *EGFR* status in the primary loci; E20 ins, insertion in exon 20; Del in E19, deletion in exon 19; MP, micropapillary; LN, lymph node; –, not metastasized or tissue unavailable from patient^*^^#^50; WT, wild type; ^#^patient was not involved for further analysis.
